# Kaempferol Facilitated Extinction Learning in Contextual Fear Conditioned Rats via Inhibition of Fatty-Acid Amide Hydrolase

**DOI:** 10.3390/molecules25204683

**Published:** 2020-10-14

**Authors:** Hammad Ahmad, Khalid Rauf, Wahid Zada, Margaret McCarthy, Ghulam Abbas, Fareeha Anwar, Abdul Jabbar Shah

**Affiliations:** 1 Department of Pharmacy, COMSATS University Islamabad, Abbottabad Campus, Khyber Pakhtunkhwa 22060, Pakistan; hammad_ahmad_raja@hotmail.com (H.A.); khalidrauf@cuiatd.edu.pk (K.R.); wahidpharmacist@gmail.com (W.Z.); 2 Department of Pharmacology, University of Maryland School of Medicine, Baltimore, MD 21201, USA; MMcCarthy@som.umaryland.edu; 3 Department of Pharmacology, Faculty of Pharmacy, Ziauddin University, Karachi 75000, Pakistan; ghulam.abbas@hotmail.com; 4 Riphah Institute of Pharmaceutical Sciences, Riphah International University, Lahore Campus, Lahore 54000, Pakistan; fareeha.anwar@riphah.edu.pk

**Keywords:** endocannabinoids, contextual fear conditioning, fatty-acid amide hydrolase, fear extinction, kaempferol

## Abstract

**Background:** Fear, stress, and anxiety-like behaviors originate from traumatic events in life. Stress response is managed by endocannabinoids in the body by limiting the uncontrolled retrieval of aversive memories. Pharmacotherapy-modulating endocannabinoids, especially anandamide, presents a promising tool for treating anxiety disorders. Here, we investigated the effect of kaempferol, a flavonoid, in the extinction of fear related memories and associated anxiety-like behavior. **Methods:** The ability of kaempferol to inhibit fatty-acid amide hydrolase (FAAH, the enzyme that catabolizes anandamide) was assessed in vitro using an enzyme-linked immunosorbent assay (ELISA) kit. For animal studies (in vivo), the extinction learning was evaluated using contextual fear conditioning (CFC, a behavioral paradigm based on ability to learn and remember aversive stimuli). Furthermore, an elevated plus-maze (EPM) model was used for measuring anxiety-like behavior, while serum corticosterone served as a biochemical indicator of anxiety. Lastly, the interaction of kaempferol with FAAH enzyme was also assessed in silico (computational study). **Results:** Our data showed that kaempferol inhibited the FAAH enzyme with an IC_50_ value of 1 µM. In CFC, it reduced freezing behavior in rats. EPM data demonstrated anxiolytic activity as exhibited by enhanced number of entries and time spent in the open arm. No change in blood corticosterone levels was noted. Our computational study showed that Kaempferol interacted with the catalytic amino acids (SER241, PHE192, PHE381, and THR377) of FAAH enzyme **Conclusion:** Our study demonstrate that kaempferol facilitated the extinction of aversive memories along with a reduction of anxiety. The effect is mediated through the augmentation of endocannabinoids via the inhibition of FAAH enzyme.

## 1. Introduction

Stress is a physiological response to any stimulus (real or un-real) that implies a challenge to bodily homeostasis. The normal stress response helps adapt to changing external milieu [1]. However, if this response is disproportional and inappropriate in its intensity against stimuli, a maladaptive response is produced leading to exaggerated fear and anxiety-like states [2]. Fear is a crucial part of the acute stress response. Since exaggerated fear is the driving force for anxiety-like disorders, therefore, the deciphering of neuro-circuitry of anxiety and fear in animal paradigms has been extensively studied including startle, contextual fear conditioning and passive avoidance models [3]. Pathological fear and anxiety can potentially develop a number of psychological illnesses including various types of phobias, panic disorder, generalized anxiety (GAD), and post-traumatic stress disorder (PTSD) [4,5].

Brain structures involved in the emotional processing of fear or aversive memories are the limbic cortex, hippocampus, and amygdala [6]. The amygdala is an important player of limbic system, which processes emotional aspects of stress stimuli and initiate appropriate behavioral response. It is not only accountable for the expression of fear and aggressive response but also initiates specific defensive behavior [7]. An important player in stress is the endocannabinoid system (eCB) [8]. The eCB network is present in stress, anxiety, and fear-associated brain areas and constantly regulates processing of aversive memories [9]. Endocannabinoids are lipids based retrograde neurotransmitters, including anandamide (*N*-arachidonoyl ethanolamide, AEA) and 2-AG (2-arachidonoylglycerol). Two different G-protein coupled receptors of this system are identified and cloned so far in humans, i.e., CB1 and CB2 [10]. A family of prominent hydrolytic enzymes including monoacylglycerol lipase (MAGL) and fatty-acid amide hydrolase (FAAH), which degrade 2-AG and AEA, respectively, regulates the duration of action of eCB molecules [11]. The eCB system helps in regulation of complex circuitry that plays central role in anxiety-like states and is a critical component of emotional learning. Dys-regulation of eCB system has been associated with several psychiatric disorders [12]. Evidence suggests that enhancement of eCB signaling may serve as a novel treatment strategy for stress- and anxiety-related disorders. The animal models of chronic stress show a notable decrease in AEA levels in all areas of brain [13]. Likewise, inhibition of AEA metabolism was also reported to occur in anxiety [14]. THC (tetrahydrocannabidol) microinjected into the ventral hippocampus or PFC (prefrontal cortex) of rats demonstrated anxiolytic effect in elevated plus-maze. Similarly, microinjection of methanandamide (AEA analog) into the PFC of rats also produced an anxiolytic effect [15,16]. Genetic as well as pharmacologic approaches strongly support the idea that FAAH knockout mice and the wild-type administered with FAAH inhibitor (URB597) showed reduced anxiety-like behavior upon exposure to light-dark and elevated plus-maze (EPM) tests. This effect was abolished with the administration of rimonabant, the CB1 receptor antagonist. It is quite evident from this experiment that FAAH inhibition declines AEA degradation, which via CB1 modulation decreases anxiety-like behaviors [17]. Another study revealed that the FAAH inhibitor (PF-3845) reduced marble burying behavior (predictive of stress and anxiety) at doses that did not alter locomotion [18]. Furthermore, the FAAH modulation was also reported to affect the biochemical stress response as URB597 (FAAH inhibitor) administration caused significantly reduced release of corticosterone following restraint stress [19]. The FAAH inhibitor locally administered in the BLA (basolateral amygdala) was reported to prevent the decline in amygdalar AEA levels and attenuated stress-induced HPA axis activation [20]. Hence, eCB signaling regulates stress response systems, in a positive fashion by reducing stress and anxiety [21,22]. This data clearly supports the idea of targeting catabolic enzymes of eCB system to develop new classes of anxiolytics [18].

Flavonoids are the subclass of polyphenol compounds with tremendous therapeutic potential [23]. They have been previously found to interact with FAAH and eCB system [24]. Daidzein, found in alfalfa roots, has capacity to inhibit FAAH enzyme in rat brain [25]. Similarly Isoflavonoids, 7-hydroxyflavon and biochanin A demonstrated potent FAAH inhibitory activity both in vitro and in vivo [26]. These findings suggest that flavonoids can possibly inhibit FAAH and modulate eCB system. Kaempferol, a natural flavone, is found in many plants (beans, broccoli, cabbage, gooseberries, grapes, kale, strawberries, tomatoes, citrus fruits, Brussels sprouts, apples, and grapefruit) and has been researched for its wide range of therapeutic actions. It is reported to be particularly effective against ailments involving inflammation [27], cancer [28], and oxidative stress [29] through modulation of molecular pathways such as NF-kB, PI3k/AKT, MAPK, Bcl2, Caspase 3, and VEGF [30]. It is of note that kaempferol is capable of exerting beneficial effects against central nervous system disorders as well, such as depression [31], anxiety [32], and cognitive deficit [33]. Keeping in view the aforementioned reports, the present study was designed to observe the effect of kaempferol on fear memory processing and associated anxiety in the context of eCB modulation.

## 2. Results

### 2.1. FAAH Inhibitory Activity

**Figure 1:**
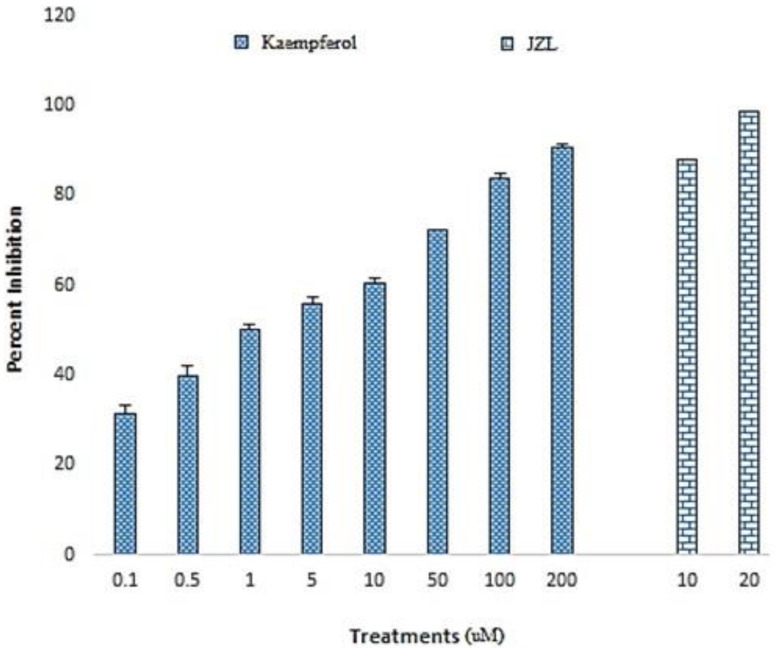
Inhibition of the FAAH enzyme by kaempferol. The figure depicts the dose-dependent inhibition of FAAH by kaempferol (0.1–200 µM) and standard JZL165 (10 and 20 µM). Data are shown as the mean ± S.E.M of percent inhibition.

Kaempferol inhibited FAAH in a dose-dependent fashion with an IC_50_ value of 1.064 µM (Figure 1). Moreover, the 200 µM dose of kaempferol (90.79 ± 1.11%) produced an equivalent response to a standard FAAH inhibitor, i.e., JZL195 10 µM (88.02 ± 1.26%).

### 2.2. Contextual Fear Conditioning (CFC)

**Figure 2:**
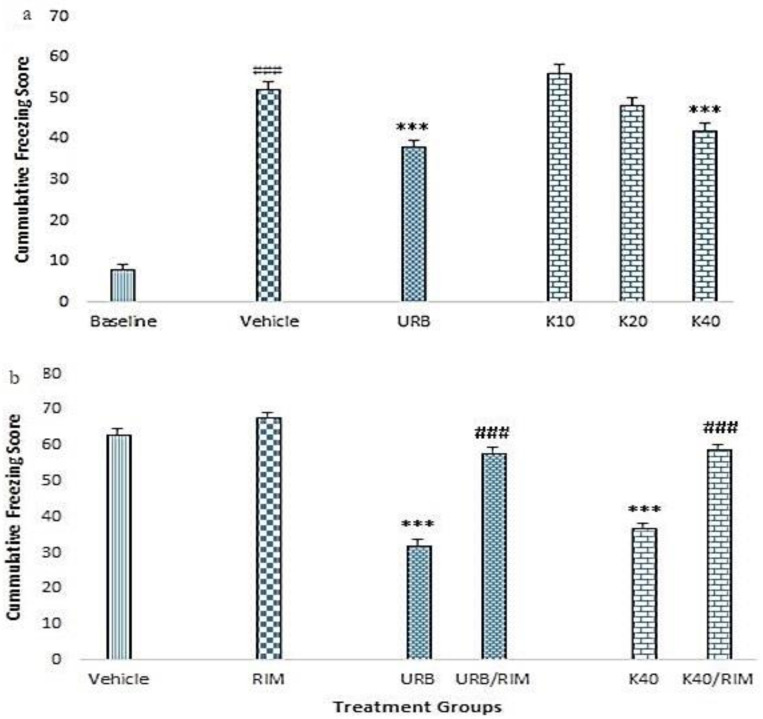
Effect of kaempferol on the cumulative freezing scores of fear-conditioned rats. Rats were acclimatized for three days and baseline-freezing behavior was recorded by placing the rats in a fear- conditioning cage. (**a**) Depiction of the freezing score in the presence of various treatments, i.e., vehicle, (URB597 1 mg/kg) and kaempferol (K10, 20 or 40 mg/kg). The *** indicate *p* < 0.001 as compared to vehicle control. The ### shows *p* < 0.001 as compared to baseline. (**b**) Depiction of the effect various treatments in the presence of rimonabant (RIM, a CB1 receptor antagonist, 1 mg/kg). The freezing scores during extinction sessions were compared among various treatment groups. *** *p* < 0.001 as compared to vehicle. ### *p* < 0.001 as compared to respective control (URB or K40 alone) without RIM. Data are shown as the mean ± S.E.M of cumulative freezing of rats for five days.

Our data showed (Figure 2a) that the rats subject to fear conditioning exhibited significantly higher freezing scores as compare to baseline groups (F _(1,58)_ = 87, *p* < 0.001). The standard drug URB treatment caused a significant decline in the freezing score as compared to vehicle control (F _(1,98)_ = 26, *p* < 0.001). Furthermore, the kaempferol-treated rats showed decreasing trend of freezing score, which became significant at the highest tested dose of 40 mg/kg [F _(4,245)_ = 13, *p* < 0.001].

In another set of experiment, the rimonabant treatment did not significantly alter the freezing score of rats as compared to vehicle control (F _(5,293)_ = 101, *p* > 0.05) (Figure 2b). On the contrary, significantly lower freezing scores were noted for URB, and kaempferol 40 mg/kg (*p* < 0.05 for both respectively), when compared to vehicle control. Furthermore, the pre-treatment with rimonabant caused a significant reversal of the freezing scores obtained with URB (F _(3,196)_ = 106, *p* < 0.001) and kaempferol 40 mg/kg (F _(3,196)_ = 93, *p* < 0.001) (Figure 2b).

### 2.3. Elevated Plus Maze Test

**Figure 3:**
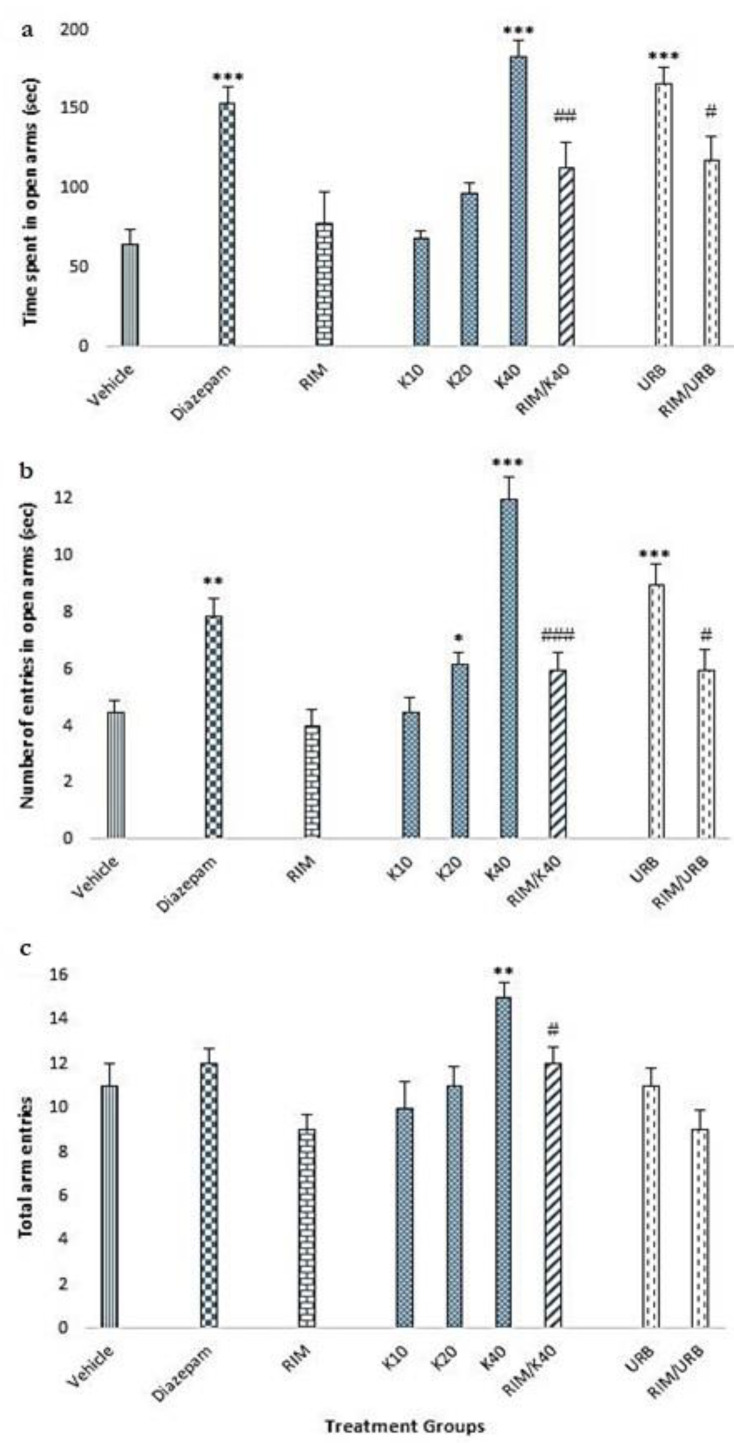
Effect of kaempferol on the levels of anxiety in elevated plus maze. After CFC, rats were subjected to EPM and parameters were noted: (**a**) Time spent in open arms. (**b**) Number of entries in the open arms. (**c**) Total arm eateries. Diazepam was used as standard. * *p* < 0.05, ** *p* < 0.01, and *** *p* < 0.001 as compared to vehicle control. # *p* < 0.05, ## *p* < 0.01, and ### *p* < 0.001 as compared to respective controls (URB or K40 alone), i.e., without CB1 antagonist RIM; 10, 20, 40 (kaempferol 10, 20, or 40 mg/kg), URB (URB597 1 mg/kg), RIM (rimonabant 1 mg/kg).

Our data showed that the standard diazepam treatment caused a significant increase in the time spent in the open arms of elevated plus maze as compared to vehicle control (F _(8,63)_ = 12.7, *p* < 0.001) (Figure 3a). In similar lines, kaempferol 40 mg/kg (*p* < 0.001) and URB (*p* < 0.001) also caused significant increases in the open arm time. Additionally, the co-administration of rimonabant caused a significant reversal of the effect produced by kaempferol 40 mg/kg (F _(1,14)_ = 14.4, *p* < 0.01) and URB (F _(1,14)_ = 7, *p* < 0.05) on the time spent in the open arms (Figure 3a).

Additionally, significant increase [F _(8,63)_ = 15.8] was observed in the number of open arm entries in rats treated with kaempferol 20 mg/kg (*p* < 0.05) and 40 mg/kg (*p* < 0.001), diazepam (*p* < 0.01) and URB (*p* < 0.001) as compared to vehicle control (Figure 3b). Additionally, the co-administration of rimonabant caused a significant reversal of the effect produced by kaempferol 40 mg/kg (F _(1,14)_ = 35.5, *p* < 0.001) and URB (F _(1,14)_ = 4.7, *p* < 0.05) on the number of entries in the open arms (Figure 3b).

Among various treatments and its combinations, only kaempferol at the dose of 40 mg/kg caused a significant elevation in the total arm entries of elevated plus maze as compared to vehicle control [F _(8,63)_ = 3.9, *p* < 0.01] (Figure 3c). Additionally, the co-administration of RIM caused a reversal of the effect produced by kaempferol 40 mg/kg and URB on the total arm entries but statistical significance was achieved with kaempferol 40 alone (F _(1,14)_ = 8, *p* < 0.05).

### 2.4. Corticosterone Levels

**Figure 4:**
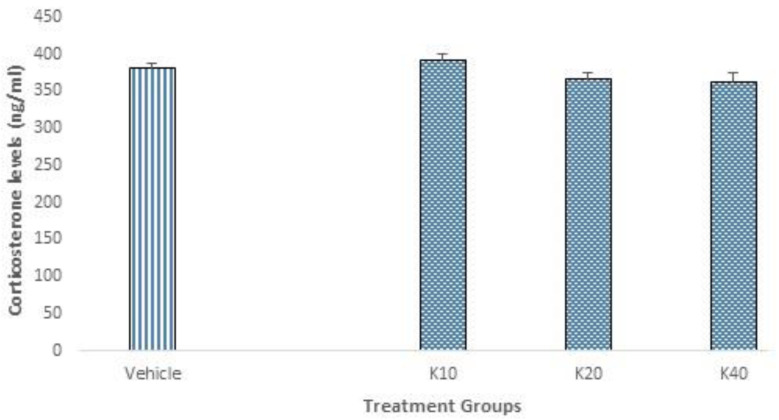
Effect of kaempferol on serum corticosterone levels. After CFC, corticosterone was measured in the serum using an ELISA kit. No significant change was noted. Data is represented as mean ± S.E.M. K10, 20, 40 (kaempferol 10, 20 or 40 mg/kg).

Serum corticosterone levels remained unaltered in kaempferol-treated animals as compared to vehicle control (Figure 4).

### 2.5. In Silico Study

**Figure 5:**
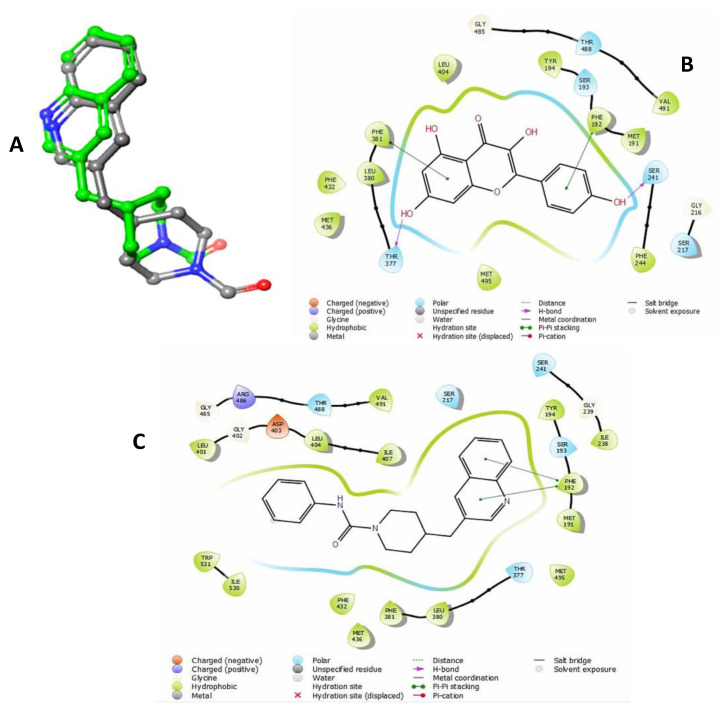
In silico docking analysis of kaempferol. (**A**) Rigid receptor docking (RRD) of the co-crystalized ligand at the active site of FAAH. It shows a cognate re-docked pose (green) of kaempferol compared with co-crystalized pose (elemental). (**B**) Kaempferol interaction with residues of FAAH active site simulated in two-dimensional (2D) plot to visualize the key interacting residues. (**C**) PF-750 interaction with residues of FAAH active site simulated in 2D plot to visualize the key interacting residues.

Kaempferol was assessed by in silico modeling against the FAAH enzyme to investigate the affinity, precise binding mode, and putative interactions with the enzyme’s active site, in comparison with standard FAAH inhibitor PF-750 [34]. The binding site of FAAH consists of four vital domains. Firstly, catalytic triad that is composed of SER241, SER217, and LYS142 and is responsible for enzymatic hydrolytic activity. Secondly, an oxyanion hole containing GLY240, GLY239, SER241, and ILE238 orient the substrate for binding. Thirdly, a dynamic gate formed by PHE432 and TRP531 guide and anchor the substrate throughout catalysis. Fourthly, an entry gate for the substrate is shaped by PHE381 and ASP403 [35]. The cognate re-docking revealed that the binding of the ligand at the active site yielded a pose similar to the referenced co-crystalized pose with RMSD 1.242 Å. This validated the docking protocol (Figure 5A).

The docking simulation showed the negative binding free energies of both the ligands towards the FAAH binding pocket, and signified their inhibitory potential. The binding free energy of PF-750 served as a standard’s threshold with −54.46 Kcal/mol of ΔG_bind_ (Table 1). It was observed that the binding affinity of kaempferol was higher than standard’s threshold with −63.92 Kcal/mol of ΔG_bind_. This binding affinity was higher than the PF-750 (Figure 5C), since kaempferol could establish more contact with FAAH than the standard compound. Binding mode analysis revealed that kaempferol penetrated into FAAH’s catalytic triad to establish contact with key catalytic residues and support the catalytic potential and negative ΔG_bind_ of the compound (Figure 5B; Table 1). Kaempferol complex was stabilized by the pi-pi stacking with PHE192 and PHE381 while H-bonding was formed with SER241 and THR377 at the binding pocket of the FAAH enzyme (Figure 5B). These interactions predict the capacity of kaempferol to inhibit FAAH enzyme activity [35].

## 3. Discussion

Stress-related disorders are prevailing and significantly contribute to disease burden with serious socio-economic implications [36]. Such disorders have well established picture of organic changes in brain chemistry and associated behavioral manifestations [37]. Brain homeostasis in acute stress and anxiety involves diverse neurochemical basis. Among all, the endocannabinoid system is the prominent modulator and relieves stress and anxiety by extinction of fear-related memories through a mechanism known as extinction learning [38]. In search of the pharmacological enhancer of extinction learning, the present study was designed to evaluate the effectiveness of kaempferol as an eCB modulator using in vitro, in vivo, and in silico sets of experiments. 

Our in vitro analysis showed that kaempferol inhibited FAAH enzyme in a concentration-dependent manner with an IC_50_ value of 1.064 µM, thereby supporting its ability to augment the endocannabinoid activity (Figure 1) [39]. This is in line with the earlier report exhibiting the inhibitory potential of kaempferol against FAAH enzyme [40]. The contextual fear-conditioning paradigm was used to assess the effect of kaempferol in vivo. In our experimental design, light/tone impulse served as an emotionally neutral conditioned stimulus (CS) that was paired with a short electric foot shock, an aversive unconditioned stimulus (US). This conjures a measurable fear reaction, like freezing [41]. Once the CS–US association is memorized, a consolidated fear memory is observed in rodents where CS predicts US and, later, CS alone can elicit innate physiological and behavioral responses to context re-exposure. Repeated exposure to fear context diminishes fear response via extinction of fear memory, an inhibitory learning process that competitively overpowers the original fear memory and reaction towards it. Gradual extinction of fear response is the result of decline in predictive nature of CS and US indicating the abolition of aversive memory [42]. Like other forms of learning, extinction also occurs in three phases, i.e., acquisition (decrease in conditioned responses to the presentation of a CS without the US), consolidation (a time-dependent process during which a long-term extinction representation is formed), and retrieval. Good extinction retrieval is characterized by low levels of conditioned responses upon presentation of CS later; whereas poor extinction retrieval is characterized by high levels of conditioned responses. The latter is observed following renewal, reinstatement, spontaneous recovery, or in pathological conditions characterized by extinction failure such as in case of posttraumatic stress disorder [43]. Extinction learning and expression relies primarily on three specific structures of the brain such as amygdala, prefrontal cortex, and hippocampus. Numerous molecular mechanisms (receptors and signaling pathways) are involved in the process of extinction learning, i.e., glutamatergic, nor-ardrenergic, dopaminergic, cannabinoids, and glucocorticoid. Hence, their pharmacological modulators are considered useful as enhancers of extinction learning thereby may serve as potential lead for the management of PTSD [44]. 

Our data showed that co-administration of CS-US during the acquisition phase induced significant fear among the rats. It was expressed by cumulative freezing behavior during extinction sessions (only CS), and was significantly higher among all conditioned rats as compared to baseline freezing behavior before conditioning (Figure 2a). Five consecutive extinction sessions progressively reduced the freezing behavior among vehicle group but it failed to produce significant extinction alone. Repeated administration of CS in the absence of US, diminishes the fear associated with US by forming a new association between CS and absence of US, rather than erasing the previous CS-US memory [45]. However, formation of this new memory is subjected to variety of phenomenon like context, exposure time, intensity of trauma, and individual variability which cause extinguished fear memory recall (memory reconsolidation) simultaneously, leading to impaired extinction [46]. Treatment with kaempferol reduced the freezing response among contextual fear conditioned rats in a dose dependent manner and maximum response was observed with Kaempferol 40mg/kg that was comparable with standard FAAH inhibitor URB (Figure 2a). This showed that kaempferol ameliorated the memory reconsolidation as well as facilitated the extinction in treated groups at the given doses. Although not significantly different from standard FAAH inhibitor URB, which has been previously reported to reduce contextual fear-related freezing behavior [47], the reduction in freezing behavior with kaempferol was higher (Figure 2a). Thus, Kaempferol mediated FAAH inhibition impaired premature amalgamation of contextual fear conditioning ^48^ but also enhanced the extinction of conditioned fear-related aversive memories [48]. 

Fear conditioning exerts anxiogenic effect, which can be measured in EPM [49,50]. Pharmacologic modulation of endocannabinoids have been associated with anxiolytic effects, where FAAH inhibitors and anandamide transport inhibitors have been identified to exert anxiolytic effects via increasing endogenous anandamide levels [51,52,53,54]. In our study, kaempferol (40 mg/kg) and URB treatment showed anxiolytic effects as depicted by increase in total time spent in open arm (Figure 3a) and the frequency of open arm (Figure 3b) and total arm entries (Figure 3c). The anxiolytic effects were comparable with standard anxiolytic diazepam and significantly higher than vehicle group. It is in accordance with literature where endocannabinoid modulation by either FAAH inhibition [55] or CB1 activation [56] lessened fear and stress related rodent behavior. Similarly, increase in brain levels of anandamide by FAAH inhibitor induced anxiolytic-like effects in rodents presented to an aversive environment [52]. Moreover, search of literature revealed that kaempferol-mediated anxiolytic action is dependent on route of administration, i.e., oral is effective while intraperitoneal is not effective. Hence, kaempferol may be a prodrug producing active anxiolytic molecule upon metabolism in gastrointestinal tract [57]. However, in our study since the mode of administration was intraperitoneal therefore the possibility of kaempferol as prodrug is ruled out. 

The behavioral effects produced by kaempferol or URB treatment during CFC and EPM were abolished by co-administration with CB1 receptor antagonist rimonabant (Figure 2b, Figure 3a–c). Rimonabant at the lower dose of 1 mg/kg has been reported to antagonize exogenous modulation of cannabinoids by pharmacologic inhibition of CB1 receptors, without producing overt behavioral effects [54]. The freezing behavior was significantly high (Figure 2b), so open arm entries and time were reduced (3a,b) when kaempferol 40mg/kg and URB-treated groups were co-treated with rimonabant. This shows that the effects produced by Kaempferol and URB were CB1 receptor mediated. This is in agreement with previous data, which shows that pharmacologic augmentation of AEA by FAAH inhibition facilitated extinction learning and anxiolysis and was interrupted by blockade of CB1 receptors [17,58,59].

The neuroendocrine effects of kaempferol on fear conditioned rats subjected to five-day extinction trials were investigated by assessing the serum corticosterone levels. Our data demonstrated no change in corticosterone levels in vehicle and kaempferol treated rats (Figure 4). These results are consistent with some of the previous report where endocannabinoid modulation via FAAH inhibition or by anandamide reuptake blockade, affects emotional and behavioral aspects of stress and fear, without any significant change in neuroendocrine aspect of fear or stress response [60,61,62].

The in silico tools provide useful means to study the nature of chemical interaction(s) of chemical(s) with the pharmacological targets. Our data (computational docking simulations) demonstrated insight into orientation and energetically favorable binding pose of kaempferol with FAAH (Figure 5, Table 1). Rigid-receptor docking coupled with Prime MM/GBSA simulation highlighted kaempferol as a potential FAAH inhibitor with low binding free energy compared to standard compound PF-750 (Table 1) [34]. Interestingly, the structural scaffolds of kaempferol shares important lead with potential FAAH inhibitors where formation of H-bonding with SER241 and interaction with PHE381 may be responsible for catalytic inhibition of FAAH (Figure 5) [34,63,64,65]. 

In conclusion, the present study demonstrates that kaempferol has the potential of facilitating extinction of aversive memories along with alleviation of anxiety possibly through eCB augmentation via inhibition of the FAAH enzyme.

## 4. Methodology

### 4.1. Drugs and Chemicals

A fatty acid amide hydrolase inhibitor screening assay kit (Item No. 10005196) and JZL195 (4-[Bis(1,3-benzodioxol-5-yl)hydroxymethyl]-1-piperidinecarboxylic acid 4-nitrophenyl ester) were purchased from Cayman Chemical Company (Ann Arbor, MI, USA). The corticosterone ELISA kit (ab108821) was from Abcam Inc. (Eugene, OR, USA). Kaempferol, rimonabant, URB597, and diazepam were purchased from Sigma-Aldrich, Darmstadt, Germany.

### 4.2. Animals

Adult Wistar rats (three-months old) weighing 200–300 g were housed in standard environmental conditions in plastic cages (6 rats/cage) at 23 ± 2 °C and under a 12/12 h light/dark cycle. Animals were provided with food and water ad libitum. All behavioral tests were conducted during the light phase of the cycle. All experimental procedures of the study were performed in accordance with the guidelines mentioned in Animal Scientific Procedure act 1986 (UK) and were approved by the ethical committee (No. 17/2258), department of pharmacy, COMSATS University, Islamabad, Abbottabad campus. 

### 4.3. FAAH Enzyme Inhibition Assay

FAAH inhibition assay kit was used to measure FAAH inhibitory activity of kaempferol according to the manufacturer’s instructions. Kaempferol was tested for its activity at concentrations (0.1, 0.5, 1, 5, 10, 50, 100, and 200 µM) and JZ195 provided with the kit was used as standard FAAH inhibitor (10 and 20 µM). Results were calculated and expressed as % inhibition of FAAH [66].

### 4.4. Behavioral Studies

**Figure 6:**
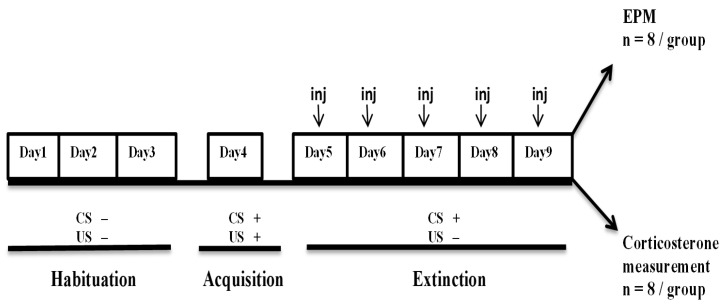
Experimental design for behavioral study.

Rats (*n* = 104) were randomly assigned to various treatment groups (*n* = 8 per group). All groups were first subjected to contextual fear conditioning (CFC) protocol along with respective drug treatments. Immediately after the completion of CFC, the animals were either subjected to the elevated plus maze (EPM) protocol and/or euthanized for serum corticosterone measurements. The diagrammatic representation of experimental design is shown in Figure 6.

The CFC protocol is divided into three phases, i.e., habituation, acquisition, and extinction. Immediately after the last extinction rats were either subjected to EPM (*n* = 8/group) or euthanized for serum corticosterone measurement. CS is conditioned stimulus, i.e., light/sound. US is un-conditioned stimulus, i.e., electric foot shock.

#### 4.4.1. Contextual Fear Conditioning

Contextual fear conditioning was accomplished following previous methods [53,67] of Pavlovian fear conditioning. The apparatus consisted of a transparent plexiglas walled chamber (22 cm × 22 cm × 25 cm) fitted with light and sound pulse sources. Floor was made up of electrifiable stainless steel grid. The CFC protocol is divided into three phases, i.e., habituation, acquisition, and extinction as described below.

##### Habituation

Before the start of conditioning trial, rats (*n* = 8 per group) were habituated to the environment for three days. For habituation (Day 1–3), rats were individually placed in the chamber for five minutes and returned to the home cages and baseline freezing behavior was recorded. Freezing is stated as the absolute immobility of rats except movements induced by breathing induced. Freezing time of the animals was recorded by a trained observer blinded with the treatment orders during the course of the study, with a stopwatch, and percent freezing was calculated. Baseline represents the mean freezing values of three days of habituation.

##### Acquisition

On the day of conditioning, animals were exposed to a light/sound pulse, i.e., conditioned stimulus (CS) followed by an electric foot shock, i.e., unconditioned stimulus (US) by placing in the chamber individually. Each animal CS-US pairings of light (2.5 s)/sound pulse (10 s; 67 db; 4 kHz tone) terminating with an electric shock (2 s; 1.5 mA). A total of 10 to ten CS-US pairings per animals were provided with is 30 seconds intervals and continued over duration of five minutes. Later, rats were placed back in their respective home cages.

##### Extinction

After 24 h of CFC, the rats were treated with either test compound kaempferol (10, 20, or 40 mg/kg), standard FAAH inhibitor URB (1 mg/kg), CB1 receptor antagonist rimonabant (1 mg/kg) or vehicle [52]. All compounds were dissolved in 0.2 mL dimethylsulfoxide (DMSO), which was diluted to the final volume with methylcellulose (0.4%) in saline. The methylcellulose (0.4%) in saline was used as vehicle [54]. Rats were subjected to aforementioned light/sound conditions (CS) without electric foot shock (US) 30 min after dosing. The whole experiment was reproduced once daily (i.e., CS without US) for five days at the same time. Results were expressed as cumulative five days freezing score of animals [53,61].

#### 4.4.2. Elevated Plus-Maze (EPM)

EPM was used to assess stress related anxiety behavior among rats [68,69]. The apparatus contained four arms (open/closed; 50 cm long; 10 cm wide) in the form of a plus shape and was raised 80 cm above the surface of floor. Closed arms were closed by 40 cm high barriers whereas open arms were without such barriers. Rats were placed in the center of maze for 5 min and the time spent in open arm (sec) and frequency of open arm and total arm entries were noted. Diazepam (2 mg/kg) was used as standard anxiolytic drug.

### 4.5. Serum Corticosterone Levels

Immediately after the last extinction trial, another lot of rats (*n* = 8/group) was euthanized and blood samples were collected to measure corticosterone levels using ELISA kit (Abcam, OR, USA), as described in the manufacturer protocol [61].

### 4.6. In Silico Analysis

Molecular docking was performed to predict the probable binding and inhibition of FAAH enzyme by kaempferol using Molecular Modeling Software with the Maestro 11.8 interface (Schrödinger LLC. 2018-4) [70].

#### 4.6.1. Protein Preparation

The three-dimensional (3D) X-ray crystallized structure of FAAH (PDB ID: 2VYA) was retrieved from RSCB Protein Data Bank (http://www.rscb.org/), and prepared in Protein Preparation Wizard using OPLS 2005 force-field (Schrödinger LLC). The structure was pre-processed to assign bond orders, add hydrogens and disulfide bridges, create zero-order bonds to metals, remove water molecules beyond 5Å of heteroatom (HET) groups, and generate HET states using Epik at pH 7.0 ± 2.0. Protein structure was refined to optimize the H-bond assignment by sampling water orientations with PROPKA at pH 7.0 as well as processed for restrained minimization to converge heavy atoms to 0.30 Å RMSD [71].

#### 4.6.2. Ligand Preparation

The 3D conformers of standard PF-750 (CID: 25154868) and kaempferol (CID: 5280863) were retrieved from PubChem database. These structures were prepared in LigPrep module (Schrödinger LLC) to produce low-energy 3D conformations using OPLS 2005 force-field [72]. The ionization and tautomeric states were generated using Epik at pH 7.0 ± 2.0. Stereoisomeric chirality was retained to original state and maximum 32 conformations were generated for the ligand.

#### 4.6.3. Molecular Docking

The rigid receptor docking (RRD) was performed with standard precision (SP) using Glide software (Schrödinger LLC). The van der Waals (vdW) radii of receptor was softened by 1.0 scaling factor with partial charge cut-off value of 0.25 and receptor grid was generated at the centroid of co-crystalized ligand for docking protocol. The vdW radii of ligands were softened to 0.80 scaling factor with partial charge cut-off value of 0.15. Ligands were flexibly sampled and post-docking minimization was performed to produce, at most, 20 poses for each ligand and ranked per Glide score (Kcal/mol). The cognate re-docking was performed to validate the docking protocol and RMSD value of co-crystalized ligand was calculated.

#### 4.6.4. Prime/MM-GBSA Simulation

The RRD complex was further processed in Prime/Molecular Mechanics Generalized Born Surface Area (Prime/MM-GBSA) simulation to accurately calculate the binding free energy (ΔG_bind_) by following equation (1) [72]:

ΔG_bind_ = ΔE_MM_ + ΔG_solv_ + ΔG_SA_

where ΔE_MM_ is the difference in minimized energy of RRD complex and Σ energies of unbounded receptor and ligand, ΔG_solv_ is the difference in GBSA solvation energy of RRD complex, and Σ solvation energies of unbounded receptor and ligand.

### 4.7. Statistical Analysis

The data is expressed as mean ± S.E.M (*n* = 8/group). The difference between various means was computed using One-way ANOVA followed by post hoc test (LSD) using SPSS 19.0. The level of significance was set at *p* < 0.05.

## Figures and Tables

**Table 1:** Parameters for in silico docking simulation and computed free binding energy by Prime/MM-GBSA.

Target	Compound	Glide Score (Kcal/mol)	Prime MM/GBSA ΔG_bind_ (Kcal/mol)	Interacting Residues	Interaction Types
FAAH	PF-750	−8.575	−54.46	PHE192	Pi–Pi stacking
	Kaempferol	−9.199	−63.92	SER241, PHE192, PHE381, THR377	H-bonding, Pi–Pi stacking
